# Regulation of Antitumor Immune Responses by Exosomes Derived from Tumor and Immune Cells

**DOI:** 10.3390/cancers13040847

**Published:** 2021-02-17

**Authors:** Andrés Rincón-Riveros, Liliana Lopez, E Victoria Villegas, Josefa Antonia Rodriguez

**Affiliations:** 1 Bioinformatics and Systems Biology Group, Institute for Genetics, Universidad Nacional de Colombia, Bogotá 111321, Colombia; warinconr@unal.edu.co; 2 Department of Statistics, Universidad Nacional de Colombia, Bogotá 111321, Colombia; llopezk@unal.edu.co; 3 Biology Program, Faculty of Natural Sciences, Universidad del Rosario, Bogotá 111221, Colombia; victoria.villegas@urosario.edu.co; 4 Research Group in Cancer Biology, Instituto Nacional de Cancerología, Bogotá 111511, Colombia

**Keywords:** extracellular vesicles, exosomes, cancer, immunology

## Abstract

**Simple Summary:**

A lot of interest has been placed into extracellular vesicles as an intercellular communication mechanism with potential clinical application. As these vesicles are found naturally in body fluids such as blood, urine and saliva, it is possible to isolate them from a liquid biopsy to analyze their content, elucidate their interaction with different cell populations (crosstalk) and determine their effect during the development of a particular pathology. Clinical applications of exosomes include the discovery of diagnostic or prognostic biomarkers for cancer and other diseases, and therapeutic approaches such as organ-specific delivery of drugs, among others. Here, we provide a specific review of how extracellular vesicles, such as exosomes, are carriers for biomolecules like immune checkpoint proteins, ligands, receptors and a wide range of RNA species, which can have an impact on cancer development.

**Abstract:**

Exosomes are lipid membrane-enclosed vesicles released by all cell types that act at the paracrine or endocrine level to favor cell differentiation, tissue homeostasis, organ remodeling and immune regulation. Their biosynthesis begins with a cell membrane invagination which generates an early endosome that matures to a late endosome. By inward budding of the late endosome membrane, a multivesicular body (MVB) with intraluminal vesicles (ILVs) is generated. The fusion of MVBs with the plasma membrane releases ILVs into the extracellular space as exosomes, ranging in size from 30 to 100 nm in diameter. The bilipid exosome membrane is rich in cholesterol, ceramides and phosphatidylserine and can be loaded with DNA, RNA, microRNAs, proteins and lipids. It has been demonstrated that exosome secretion is a common mechanism used by the tumor to generate an immunosuppressive microenvironment that favors cancer development and progression, allowing tumor escape from immune control. Due to their ability to transport proteins, lipids and nucleic acids from the cell that gave rise to them, exosomes can be used as a source of biomarkers with great potential for clinical applications in diagnostic, prognostic or therapeutic areas. This article will review the latest research findings on exosomes and their contribution to cancer development.

## 1. Introduction

During the last decades, great interest has arisen in the study of the alternative routes of cellular communication, a cell function essential for multicellular organisms to maintain homeostasis, particularly those mediated by the release of extracellular vesicles. These vesicles, secreted by most of the nucleated cells, are formed by a lipid bilayer and, according to their size, are classified as: (i) exosomes, of endocytic origin, with a diameter of 50–100 nm; (ii) microvesicles, formed by external budding of the plasma membrane, with a diameter of 100–1000 nm; (iii) apoptotic bodies, formed by membrane blebbing of an apoptotic cell, with a diameter greater than 500 nm [1].

Exosomes are nanosized vesicles released into the extracellular space upon fusion of MVBs with the plasma membrane [2]. They were first described in studies of rat reticulocyte differentiation and later in human B-lymphocytes and dendritic cells (DCs) [3]. Released exosomes can influence the activity of neighboring cells (paracrine action), travel to different sites in the body through the bloodstream (endocrine action) and be found in body fluids, such as urine, plasma, breast milk, nasal discharge and cerebrospinal fluid. Pathologically, they are found in ascites, bronchial washings and pleural effusions among others [4,5].

Initially, it was believed that extracellular vesicles were a mechanism to remove cellular waste resulting from cell metabolism or damage, but exosomes play essential roles as vehicles for intercellular communication with specific targets [2]. They were shown to carry cell-specific cargos of proteins, lipids and nucleic acids, and can be selectively taken up by neighboring or distant cells. Released exosomes, upon contact with their target cells, may be internalized by pinocytosis or by endocytic receptors and can go into the lysosome for degradation. However, they can also mediate cell- cell interactions by binding the target cell membrane and directly activating cell membrane receptors, or by reaching specific cellular compartments to release their intraluminal material, thus transferring proteins from the lipid bilayer to the membrane of the target cellular compartment [6,7].

Exosomes exhibit different interactions with the immune system. Under normal conditions, they take part in antigen presentation and immune activation, whereas, in pathologic conditions such as cancer, they act as an immune evasion mechanism during tumor development, supporting crosstalk between cancer and immune cells [8].

## 2. Exosome Biogenesis

Exosomes are generated from late endosome membrane by an unconventional inward budding that results in the creation of large MVBs that act as a sorting platform for membrane proteins to develop ILVs loaded by the endosomal sorting complex required for transport (ESCRT) [9]. ILVs may follow one of three destinations: (i) if MVBs fuse with lysosomes, they are degraded; (ii) they could contribute to the development of specialized organelles such as melanosomes [10]; (iii) if the MVBs fuse with the plasma membrane, ILVs are secreted as exosomes into the extracellular milieu [11].

Several lipids and lipid metabolizing enzymes are involved in the formation and release of exosomes. Ceramide micro domains in areas with high sphingolipid concentrations are able to bind and generate large ceramide-rich domains to promote membrane budding [12,13]. Although the mechanism by which molecules are charged in exosomes is not clear, it is known that heparan sulfate proteoglycans, especially syndecans and their cytoskeleton coupling proteins, syntenins, appear to be involved in inducing intraluminal budding of the endosome membrane. The syntenin exosomes depend on the availability of heparan sulfate, syndecans, ALG2 interaction protein X (ALIX) and the endosomal classification complex required for transport (ESCRT) [14], which regulates the germination of the membrane on the cell surface and in the late endosome. 

**Figure 1:**
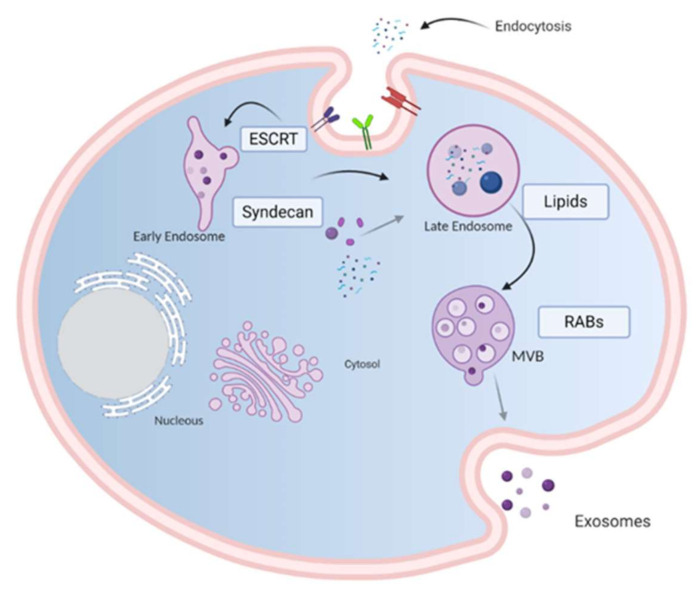
Model of biogenesis and release of exosomes. Cell-surface proteins and soluble molecules from the extracellular milieu enter cytoplasm by endocytosis or by plasma membrane invagination that results, at the luminal side of the cell, in the early-sorting endosome development which matures in a late-sorting endosome. Eventually, this late endosome generates a MVB by inward invagination of the endosomal membrane. This double invagination of the plasma membrane results in a MVB containing several ILVs. The MVBs can fuse with lysosomes where their cargo is degraded or can dock on the luminal side of the plasma membrane and merge with the plasma membrane to release the contained ILVs as exosomes. Rab GTPases, ESCRT, CD9, CD81, CD63, TSG101, ceramide, and Alix are involved in exosome biogenesis. Tetraspanins, integrins, sphingomyelinase, ceramides and immunomodulatory proteins are exosome surface proteins. The molecular exosomes’ cargo is composed of cell surface proteins, intracellular proteins, RNA, DNA, amino acids, and metabolites. Image created with biorender.com.

Loading molecules in the ILVs and exosome release from the endosomal membrane are mediated by two pathways: an endosomal sorting complex required for transport (ESCRT)-dependent pathway [15] and an ESCRT-independent pathway [16]. The ESCRT pathway comprises five different protein complexes (ESCRTs -0, -I, -II, and -III, and Vps4), capable of recognizing and classifying ubiquitinated load. ESCRT proteins are involved in the sequestration and classification of ubiquitous membrane proteins to deform the endosomal boundary membrane inward and generate the MVBs, while the Vps4 complex is needed to deliver the load to the vesicle (Figure 1) [17].

## 3. General Composition of Exosomes

**Figure 2:**
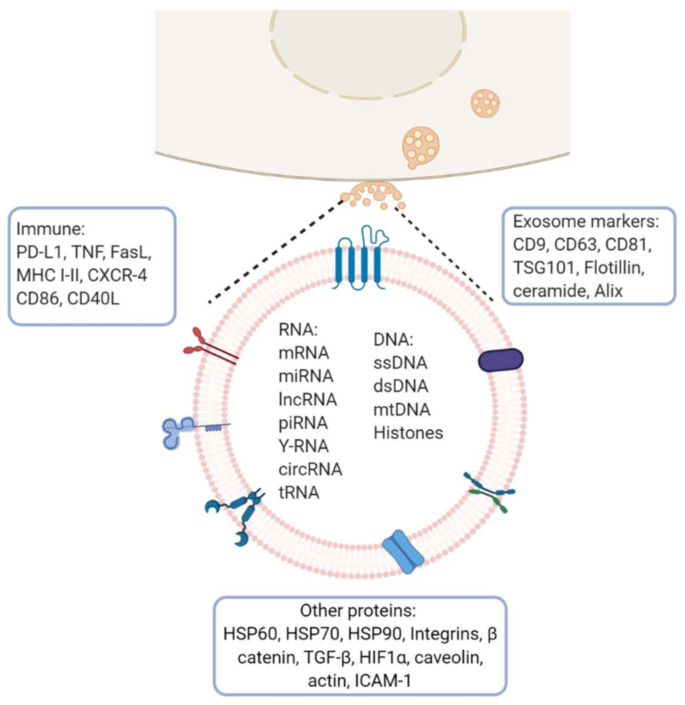
Exosome molecular cargo: Depending on their cellular origin (immune, cancer, epithelial or mesenchymal cells), exosome may have diverse molecular cargos which may include immunomodulatory proteins involved in vesicle trafficking and enzymes with functional activities in target cells. In the lumen of exosomes, they transport nucleic acids such as DNA, mRNA, miRNA, lncRNA and other RNA species. Image created with biorender.com.

Exosomes from multiple organisms harbor more than 4500 proteins, 200 lipids, 1600 mRNAs and 800 miRNAs [18]. They are present in bodily fluids and interact with adjacent cells inducing a variety of downstream effects which depend on the cell type from which they are derived and on their load-bearing molecules (membrane and cytosolic proteins, lipids, mRNA and miRNA). The composition of exosomes varies depending on the originating cell type. In general, they are surrounded by a lipid bilayer enriched with sphingomyelin, phosphatidylserine, GM3 ganglioside, phosphatidylethanolamine, MVB-specific LBPA, and GPI coupled proteins such as CD55 and CD59. Other common constituents of exosomes are tetraspanins (CD81, CD63, CD9), heat shock proteins (HSP60, HSP70, HSP90) and MHC I/II antigens. Annexins regulate cytoskeleton and cell membrane fusion, Rab proteins control the exosome secretion pathway, and GTPases favor coupling and fusion between membranes and ESCRT proteins [19,20] (Figure 2).

In general, exosome protein expression is related to the cell from which they originate, but also depends on their physiological status, e.g., exosomes released from both human and murine B-cells transformed by the Epstein-Barr virus secrete antigen-presenting vesicles capable of inducing antigen-specific MHC class II-restricted T-cell responses, suggesting a role for exosomes in antigen presentation in vivo [21]. They play a role in hepatitis B virus transmission and NK-cell dysfunction during chronic infection [22], can favor cancer progression, metastasis and drug resistance by altering gene expression in surrounding and distant cells [23], and be a source of proteins that could have clinical application as biomarkers for the early detection, diagnosis, prognosis and treatment of diseases [24].

## 4. Role of Exosomes in Antigen Presentation

Immune system modulation by exosomes can occur between cells of the same or different lineages. Effector cells (T, NK, APC, and mast cells) may give or receive information through receptor-ligand interactions or by extracellular vesicles. Tumor cell-derived exosomes (TEX) can prevent immune activation, DC maturation, T and NK cell-mediated cytotoxicity, or promote immune suppression, tolerance, and T-cell apoptosis because they can carry a large diversity of molecules to mediate intercellular communication, (Figure 1) [25,26].

As the first signal required for the activation of an effective immune response is the recognition of the HLA/peptide complexes by the T-cell receptor, and exosomes released by mature APCs express HLA/peptide complexes and costimulatory molecules necessary for T-cell activation, it seems that mature APC exosomes constitute a presentation route as crucial as that of professional APCs for T-cell activation [27,28]. In murine models, bone marrow DCs (BMDCs) have been shown to secrete exosomes capable of amplifying immune responses in vivo by transporting antigens to induce antigen-specific CD4 and CD8 T-cell activation [29,30,31]. On the other hand, regulatory activities are also carried out by exosomes. A tolerance mechanism mediated by IL-10 production, with an effect on regulatory T-cells (Treg) related to the accumulation of MFG-E8/lactadherin, has been described on exosomes from immature DCs [32,33].

Clayton et al. reported that human tumor-derived exosomes express ligands for NKG2D and TGFβ1 that triggered downregulation of NKG2D surface expression by NK and CD8(+) T-cells as an evasion mechanism to avoid their recognition and immune destruction, suggesting that NKG2D is a physiological target for exosome-mediated immune evasion in cancer [34]. The downregulation of pro-inflammatory cytokines such as IL-12p40, IL-23p19, TNF-α, and IL-1β, has also been described induced by endothelial cell-derived exosomes and the overexpression of immunosuppressive ones such as IL-10, MRC1 and TGF-β, to prevent damage and promote tissue regeneration favoring tumor growth and metastasis [35,36]. On the other hand, DCs pulsed with tumor peptides release exosomes with the ability to stimulate CD8+ T-cell proliferation and differentiation into cytotoxic T-cells. It has been also reported that exosomes can stimulate cytotoxic effects on NK cells by membrane IL-15Rα and NKG2D expression, which induce proliferation and membrane IFN production [37,38] Exosomes derived from DCs, which express major histocompatibility complex (MHC) and costimulatory molecules, have been used for antitumor vaccines because, besides to be a source of tumor peptides, they also express molecules essential for the induction of immune responses, such as MHC I, MHC II and costimulatory CD40, CD54, and CD80 [39].

NKG2D is an activating receptor for CD8+ and T-cells, NK and NKT cells. Its expression can be deregulated by its soluble ligands and by growth factors such as TGFβ1, secreted by the tumor as an evasion mechanism to prevent its recognition and immune destruction. In fact, TGFβ1 and soluble ligands for NKG2D have been detected in cancer cell lines and in tumor cells isolated from mesothelioma pleural effusions. Downregulation of NKG2D expression on NK and on the CD8+ T-cell surface [34], or the decrease in the secretion of the proinflammatory cytokines induced by these exosomes, prevents damage and promotes tissue regeneration, thus promoting tumor growth and metastasis [35,36].

Tumor-derived exosomes and micro vesicles (EMVs) are key mediators shed by cancer cells with the ability to sensitize neighboring cells in the tumor microenvironment, thus promoting cancer invasion and metastasis. EMVs derived from hypoxic tumor cells differ qualitatively from those derived from normoxic tumor cells. Hypoxic EMVs inhibit NK cell function to a greater extent than normoxic ones, by transferring TGF-β1 to NK cells, inhibiting NK cell function and decreasing NKG2D expression on the NK cell surface. They also carry high levels of miR-210 and miR-23a, which act as additional immunosuppressive factors by targeting CD107a expression in NK cells. By releasing extracellular vesicles into the hypoxic tumor microenvironment, tumor cells can educate NK cells to decrease their antitumor immune response [40].

## 5. Tumor-Derived Exosome-Mediated Immune Suppression

Exosome production by tumor cells has been implicated in cancer-associated immune suppression, and it has been proven that the body fluids of cancer patients contain large numbers of tumor exosomes (TEX) capable of downregulating the functions of immune cells and promoting tumor progression through various mechanisms including the transport of molecules such as proteins and nucleic acids. There is evidence that miRNAs, secreted by the tumor into exosomes can regulate gene expression in target cells through canonical binding to their target mRNAs. Furthermore, it has been shown that miR-21 and miR-29a secreted by the tumor can function by binding to the Toll-like receptors (TLR) family on immune cells. This binding triggers a prometastatic inflammatory response through the activation of NF-κβ, which favors tumor development and metastasis [41]. Unlike exosomes released by normal cells, TEX are involved in the regulation of peripheral tolerance. They have the ability to down-regulate CD3ζ and JAK3 expressions in primary activated T-cells, induce Fas/FasL-mediated apoptosis in TCD8+ lymphocytes, and promote CD4+CD25− T-cell proliferation and their further conversion to CD4+CD25hi+FOXP3+ Treg cells expressing IL-10, TGFβ1, CTLA-4, GrB/perforin to mediate immunosuppression [42,43].

**Figure 3:**
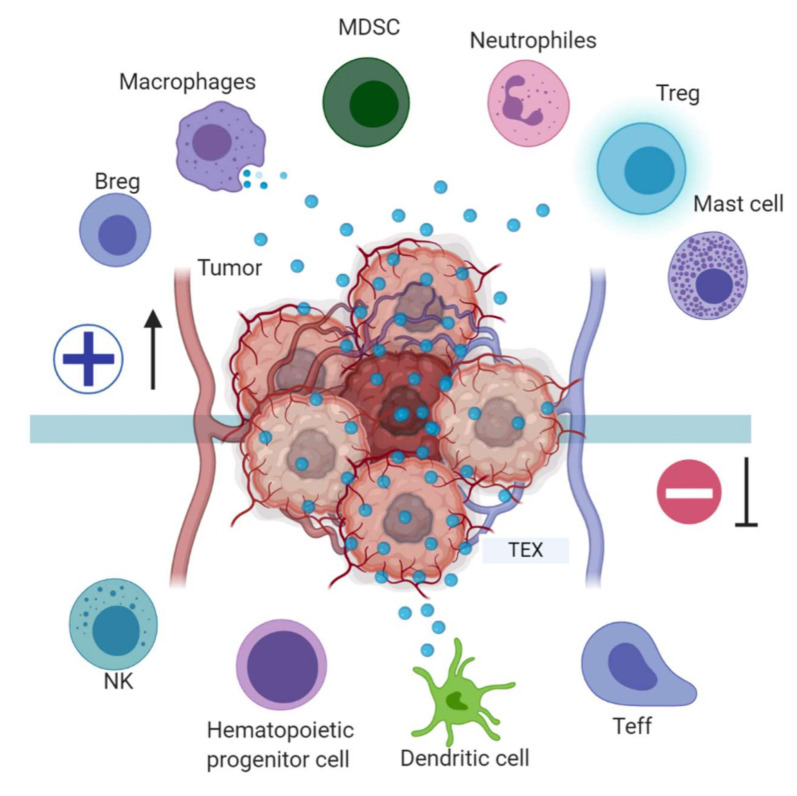
Tumor immune escape mediated by tumor exosomes (TEX). TEX favor immune escape by bearing proteins such as TGF-β, FasL, TRAIL, PD-L1, and HSP90, and miRNA such as miR-23a, miR-24-3p and miR-214, to dysregulate NK, T and B effector cells function. In dendritic cells, exosome miR-203 may downregulate TLR4 expression and increase IL-6 production, inhibiting the differentiation of myeloid precursors into DCs. TEX bearing miR-21, miR-29a, and proteins such as CSF-1, CCL2, and TGFβ may drive macrophage polarization to a M2-like phenotype and promote MDSCs differentiation contributing to cancer progression. Image created with biorender.com.

TEX, in addition to regulating the immune effector cell (TL, NK, APC) function, are also involved in blocking myeloid cell maturation to DCs through the expression of TGFβ, which induces a CD14+HLA-DR-phenotype, characteristic of myeloid derived suppressor cells (MDSC). These MDSC include DC precursors with a suppressive effect on proliferation and cytotoxic functions of tumor-specific T-cells by altering antigen processing and presentation, producing inhibitory factors such as nitric oxide and reactive oxygen species [44], and have been described in the peripheral blood of patients with oncological pathologies including hepatocellular carcinoma, bladder carcinoma, glioblastoma and multiple myeloma [45] (Figure 3). A murine model of breast cancer showed that tumor exosome induction of IL-6 expression can block bone marrow DC differentiation, and that pancreatic cancer-derived exosomes can regulate the expression of TLR4 and cytokines, such as TNFα and IL-12 expression in DCs through miR-203 [46,47].

## 6. Exosome-Mediated Crosstalk between Tumor and Immune Infiltrating Cells

Cancer-associated immune suppression contributes to cancer progression and is related to poor prognosis. There is evidence of the wide variety of molecular and cellular mechanisms used by the tumor to evade the host antitumor immune response, and the impact these mechanisms have on the maintenance of immune homeostasis and the response to immunotherapies aimed at activating the antitumor immune response.

It was reported that microvesicles and exosomes released by tumor cells contribute to cancer immunosuppression by inducing, in T-cells, the transition from the effector phenotype (CD4+CD25−) to the regulator (CD4+CD25+Foxp3+) phenotype, its clonal expansion, and activation. Interactions of tumor extracellular vesicles and exosomes with Treg cells represent a mechanism involved in the regulation of peripheral tolerance, since tumor infiltrating and peripheral blood circulating Treg cells are capable of inducing systematic effector T-cell apoptosis favoring the tumor escape from immune surveillance and are associated with a poorer prognosis. Thus, depletion of Treg cells before immunotherapy could increase the success of effector T-cell based immunotherapies [48].

TEX regulate Treg suppressor function and increase resistance to apoptosis by inducing expression of FasL, IL-10, TGF-β1, CTLA-4, granzyme B and perforin [49,50]. In cancer cell lines (bladder, colorectal, prostate and breast) exosomes expressing CD39 and CD73 ectonucleotidases have been described that can indirectly modulate the effector immune cells activity in the tumor environment as a mechanism of Treg cell-mediated immune regulation, [51]. In this case, the extracellular adenosine generated by exosomes expressing CD39 and CD73 interferes with antitumor immune responses, and thus, CD39 and CD73 inhibition effects have been studied for their possible clinical application against cancer [52,53]. It has also been reported that tumor cells produce miRNAs that are loaded into exosomes and secreted to favor the evasion of the immune response. One of these miRNAs, miRNA-214, is overexpressed in several types of human cancer and in murine models, promoting the expansion of Treg cells that produce high levels of IL-10 [54]. It was reported that tumor cells resistant to gefitinib could transfer this resistance to sensitive tumor cells in non-small cell lung cancer, thus suggesting that this effect is due to the miR-214 transfer in exosomes [55].

B-cells are important players in the tumor-induced immune response, and they constitute the second most abundant population of tumor-infiltrating lymphocytes. However, there is a population of regulatory B-cells (B-reg) that favor tumor development through IL-10, IL-35, TGF-β and IL-21 production, which prevent the expansion of proinflammatory cells [56]. There is evidence that Breg cells interfere with the antitumor response by mechanisms such as lymphotoxin production, which promotes tumor growth by inducing angiogenesis [57]. B-cells contain a specialized late endosomal compartment that hosts newly synthesized HLA-II molecules. Exosomes secreted by B-cells are capable of presenting antigens and inducing CD4 T-cell antigen-specific responses [58].

Tumor-derived extracellular vesicles can affect the function of B-cells in different ways, inducing antibodies production which, although capable of activating immune responses, do not have the antigenic potential of tumor-derived HLA-I binding peptides to activate the CD8+ T-cells. However, when these low antigenic antibodies bind the Fcγ receptors on myeloid cells, they induce their differentiation towards MDSC capable of suppressing the CD4+ and CD8+ T-cells antitumor immune responses [21], inducing differentiation of naive B cells into TGF-β-producing regulatory B cells with immune suppressor functions on CD8+ T-cells proliferation [59], or inducing IL-10 production, which also promotes the generation of regulatory B-cells capable of inhibiting T-cell activity [60].

Although the restoration of normal lymphocyte homeostasis is probably the most important component of an adequate response to immunotherapy, the role of other types of immune cells infiltrating the tumor cannot be ignored. Macrophages constitute one of the most abundant cell populations in the innate antitumor immune response. In the tumor microenvironment, there is cross-communication between the tumor, immune cells that infiltrate the tumor and normal tissue cells. It has been shown that invasion induced by tumor-associated macrophages (TAM) requires the positive regulation of Wnt5a to activate Wnt signaling, independent of β-Catenin in breast cancer cells, increasing their invasive capacity. In MCF-7 cell supernatants, it has been observed that microvesicles and exosomes occur with the ability to induce overexpression of Wnt5a in TAMs and transfer it to tumor cells, to improve their invasiveness [61]. In addition, proteomics studies have shown that in ovarian cancer, exosomes derived from TAMs can inhibit endothelial cell migration by interfering with the miR-146b-5p/TRAF6/NF-kB/MMP2 pathway. However, tumor cell-derived exosomes can transfer long noncoding RNAs (lncRNA) to reverse the TAMs’ effect on endothelial cells [62]. In gastric cancer, TAM-derived exosomes miR-21 transfer confers resistance to cisplatin [63].

**Figure 4:**
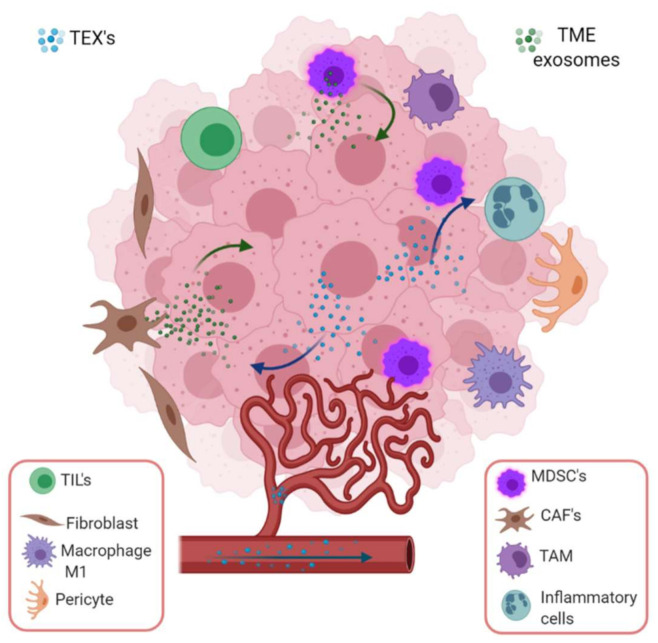
Tumor-derived exosome mediated intercommunication among stroma and tumor cells in a specific microenvironment. TEX induce epithelium mesenchymal transition (EMT), invasiveness, migration, proliferation and stemness in stroma (endothelial cells, fibroblasts, pericytes) and immune cells. In the tumor microenvironment, the most important cargoes of exosomes are HIF-1α, matrix metalloproteinase such as MMP-13, MMP-14, and ncRNAs such as miR-21, miR-135b and lncRNA-UCA1, which stimulate EMT. On the other hand, stromal cell-derived exosomes also boost cancer cells employing ncRNA (miR-21, miR-221, miR-222, miR-223, miR-100) or proteins (apolipoprotein E, Wnt, NOTCH3, TGFβR, KRAS, TNFα). Image created with biorender.com.

Some normal cells can also participate in cancer development and progression by exosome mediated mechanisms. Fibroblasts, due to their plasticity and adaptability, are able to survive in severe stress environments and may contribute to cancer development. Cross talk between cancer-associated fibroblasts (CAF) and tumor cells favor endothe-lial cells and pericytes recruitment to the tumor and stimulates the development of an appropriate tumor microenvironment (TME) (Figure 4). They can introduce metabolic and immune changes in the tumor niche to favor angiogenesis, extracellular matrix remodeling, metastasis, and escape from the immune recognition and destruction through the secretion of exosomes and proteins such as growth factors, chemokines and extracellular matrix [64,65,66].

New biomarkers associated with the promotion of growth, migration, metastasis and resistance to drugs with potential clinical application in CAF-derived exosomes, were identified [67]. It was also demonstrated that CAF secreted exosomes can affect the tumor phenotype and, in turn, exosomes released by tumor cells can activate CAF [68], as it occurs in ovarian cancer, where exosomes released by tumor cells prepare the microenvironment at a distance to favor metastatic invasion by regulating intercellular communication between tumor cells and normal stromal cells, CAF and immune cells that infiltrate the tumor [69]. Hepatocellular carcinoma (HCC) cells with high metastatic potential exhibit a more remarkable ability to convert normal fibroblasts to CAF than HCC cells with low metastatic potential, suggesting that communication between tumor cells and fibroblasts is mediated by tumor-derived exosomes that control pulmonary metastasis in HCC, and thus could be considered as potential targets for the prevention and treatment of cancer metastases [54].

## 7. Exosomes and Epithelial-Mesenchymal Transition (EMT)

Invasion and metastasis are acquired capacities of tumors characterized by the acquisition of mesenchymal cell phenotypes by epithelial tumor cells. These cells, with motile capacity, trigger the formation of metastasis in organs distant from the primary tumor [70]. The epithelial-mesenchymal transition is a reversible cellular program that occurs in normal physiological processes such as embryonic development and wound healing. During this process, cell-cell and extracellular matrix-cell interactions are remodeled with the detachment of epithelial cells from the basement membrane and the activation of transcriptomic profiles stimulated by stromal components of the tumor tissue [71]. Exosomes from the serum of patients with cancer exhibit high miRNAs expression associated with migration and invasiveness of cancer cells. miR-222 is overexpressed in lymphatic metastasis of breast cancer patients conferring a more aggressive phenotype both in vitro and in vivo. In estrogen receptor-positive breast tumors, miR-222 has been associated with high histological grade, high Ki67 proliferation rates and HER2 amplification, as well as resistance to hormonal treatment through EMT activation [72]. The primary anatomical location of metastases with the worst prognosis in gastric cancer is peritoneal metastasis whose molecular characterization and understanding is not clear. Shen et al. reported the differential expression of miR-196, miR-92 and miR1307 in ascites-derived exosomes of gastric cancer patients, with the activation of cancer-associated fibroblasts in the peritoneum [73]. Serum exosomes from patients with glioblastoma exhibit high miR-148a expression associated with migration and proliferation in an in vitro model in which gene suppression reduced proliferation and metastasis in glioblastoma cells (Table 1).

Tumor derived exosomes are one of the main strategies used by tumors to promote cancer development and metastatic spread. Via exosomal transport, they may induce new blood vessel formation by transporting vascular endothelial growth factor (VEGF), fibroblast growth factor (FGF), platelet-derived growth factor (PDGF), essential fibroblast growth factor (bFGF), tumor necrosis factor-alpha (TNFα) and interleukin-8 (IL-8), among others, to favor angiogenesis and metastasis, as well as EMT [74]. Long noncoding RNAs (lncRNA) participate in angiogenesis development of various types of cancer. lncRNA-H19 can be transported in exosomes and received by endothelial cells which promote cell adhesion and angiogenesis in a liver cancer model [75]. The long intergenic noncoding RNA-POU3F3 (Linc-POU3F3) is overexpressed in hepatocellular carcinoma [76], colorectal cancer and glioma [77], promoting tumor cell survival, proliferation, migration and invasion mediated by TEX-educated cells. The LncRNA MALAT1, associated with lung cancer metastasis, was described in ovarian adenocarcinoma as a promoter of angiogenesis and progression, [78]. 

## 8. Current Methods and Applications for Exosome-Based Cancer Therapies

The potential application of exosomes in the clinic is still emerging, and studies on their utility in the diagnosis and the treatment of many pathologies have increased during the past years. TEX represents a rich source of tumor antigens, genetic material, and immune stimulatory molecules [96]. Exosome clinical applications are not limited to their use as a source of biomarkers for diagnosis, prognosis or follow-up. Their unique capacity to transport functional cargos such as proteins and nucleic acids could be exploited using them as therapeutic vehicles to target cancer therapies to specific places with difficult access, such as the brain [97,98]. Additionally, as exosomes have the ability to stimulate specific antitumor immune responses, they also have potential application in developing cancer vaccines [99].

Exosomes are being actively explored as therapeutic agents because, in contrast to liposomes, they can deliver the molecular cargo without immune clearance [100] and have shown to be well tolerated [101,102]. Doxorubicin and other chemotherapeutic compounds such as paclitaxel loaded into exosomes for cancer therapy demonstrated antitumor efficacy and low toxicity when tested in mice [103,104]. miRNA or siRNA, administered in exosomes are protected from the ribonucleases action and could exert their function at distant sites in murine cancer models [105,106] Table 2.

Cancer-derived exosomes transport mRNAs, receptors for pathological growth factor and soluble or membrane proteins with immune checkpoint activity such as HLA-G, to support tumor spread and survival. Increased levels of exosomes in liquid biopsies of cancer patients are associated with chemotaxis and chemotherapy resistance acquisition by cancer cells increasing an active drug efflux. The above highlights the importance of finding exosomes release inhibitors to improve the antitumor effect of chemotherapy [125,126,127]. exosome and microvesicles release inhibitors such as Chloramidine/Bisindolylmaleimide, and inhibitors of neutral sphingomyelinase like GW4869, proved to be effective to enhance cancer chemotherapy efficacy [126,128,129]. However, exosome release inhibition by cannabidiol was more potent than Cl-amidine alone, but when were administered in combination, the inhibitory effect is significantly higher [130], suggesting that the anticancer activity of CBD may partly be due to its regulatory effects on the biogenesis and release of exosomes and micro vesicles by cancer cells, and that may have a clinical application to increase the efficacy of cancer chemotherapy.

## 9. Conclusions

For many decades, cancer studies focused on the tumor cell without taking into account the participation of other actors involved in carcinogenesis, such as the immune infiltrate, the population of normal cells within the tumor and extracellular vesicles [131]. The majority of nucleated cells secrete exosomes which participate in numerous biological cell processes in biological fluids. However, under pathological conditions, they can be involved in tumor development. Exosomes transport proteins, nucleic acids, lipids and different RNA species, especially noncoding RNA, capable of modulating gene expression in target cells (Figure 2). The immune system functions as a switch that turns on and off cell activity, stimulating the repression of cytotoxic T-cell functions or stimulating the proliferation and effector functions of regulatory cells. More and more studies have found that exosomes transmit biological information by transporting different RNA species, proteins, metabolites and other substances and, therefore, they could exert biological and therapeutic effects [132]. Although the therapeutic potential of exosomes has not been widely explored, innovative strategies aimed to inhibit tumor progression could contemplate systemic exosome depletion, and others are developing exosome-loaded drug delivery systems that may be applicable shortly [66].

In our review, we exposed some examples of how tumor-associated exosomes are involved in angiogenesis and in the epithelial-mesenchymal transition, the involvement of noncoding RNA and proteins contained in exosomes, and the potential use of these vesicles as a weapon to fight against this disease. Understanding the biology of exosomes may have significant implications for the diagnosis, prognosis and treatment of cancer, with great potential as biomarkers of this disease.

## Figures and Tables

**Table 1:** Biomolecules contained in exosomes and their effect on angiogenesis and metastasis.

Affected Process	Source of Exosomes	Molecular Cargo	Role in Cancer Development	Ref.
Angiogenesis	Gastric cancer patients (GC)	miR-155	Exosome miR-155 inhibit FOXO3a expression promoting angiogenesis and progression in GC. Potential biomarker for migration, angiogenesis, and poor prognosis.Novel target for anti-angiogenesis therapy	[79]
			Exosome miR-155 downregulate c-MYB and upregulate VEGF expression promoting growth, angiogenesis and metastasis in GC.	[80]
	Nasopharyngeal cancer (NPC)	miR-17-5p	Exosome miR-17-5p promotes angiogenesis in NPC though inhibition of BAMBI expression and regulation of AKT/VEGF-A signaling.	[81]
		Mir-23a	Overexpression of Exosome miR-23a in NPC promote angiogenesis in vitro and in vivo by targeting the testis-specific gene antigen (TSGA10), an angiogenesis inhibitor.	[82]
	Melanoma	miR-155	Overexpression of Exosome miR-155 in melanoma reprogram fibroblasts into CAFs by targeting SOCS1, which activates JAK2/STAT3 signaling pathway and increase the expression levels of VEGFa, FGF2, and MMP9 in fibroblasts promoting angiogenesis.Potential therapeutic target to inhibit melanoma angiogenesis.	[83]
	Ovarian carcinoma (OC)	miR-205	Exosome miR-205 from OC cells promote metastasis by inducing angiogenesis in vitro and in vivo via the PTEN-AKT pathway. Potential therapeutic target for OC.	[84]
	Non-small cell lung carcinoma (NSCLC)	miR-619-5p	Exosome miR-619-5p promotes NSCLCs growth and metastasis by regulating RCAN1.4, a tumor suppressor protein.	[85]
	Chondrosarcoma	lncRNA RAMP2-AS1	Exosome lncRNA RAMP2-AS1 from chondrosarcoma cells promotes angiogenesis proliferation, migration and tube formation favoring by the RAMP2-AS1/miR-2355-5p/VEGFR2 axis in human umbilical vein endothelial cells (HUVEC).	[86]
Metastasis	Gastric cancer (GC)	miR-27a	Exosome miR-27a from GC cells induce fibroblasts reprogramming into CAFs, promoting proliferation, motility, and metastasis in vitro and in vivo, and over-expression of miR-27a on CAFs derived exosomes could increase the malignant behavior of GC cells. miR-27a also downregulate CSRP2, increasing GC cells proliferation and motility.	[87]
	Oral squamous cell carcinoma (OSCC)	miR-34a-5p	Exosome miR-34a-5p from OSCC binds to AXL to inhibit the tumorigenesis suppressing tumor cell proliferation and metastasis. However, miR-34a-5p in CAF-derived exosomes was significantly reduced and by this mean, miR-34a-5p/AXL axis may increase aggressiveness in oral cancer cells by inducing EMT to promote metastasis by the AKT/GSK-3β/β-catenin/snail signaling which activate MMP-2 and MMP-9 expression.	[88]
	Breast cancer (BC)	miR-181d-5p	CAF-derived exosomes can transfer miR-181d-5p to enhance the aggressiveness of breast cancer by promoting tumor growth via downregulation of CDX2 and HOXA5 to favor cell proliferation, invasion, migration and EMT.	[89]
	Hepatocellular carcinoma (HCC).	TGF-β	Exosomes derived from hepatocellular carcinoma cells promote migration and invasion of recipient cells by decreasing E-cadherin expression, increasing vimentin expression and promoting EMT via TGF-β/Smad signaling.	[90]
		MALAT1	Anti-angiogenesis therapies appear to be useful for the treatment of HCC, although metastasis may develop over time. Increased expression of YAP1 around tumor-associated blood vessels indicates a role in angiogenesis for this protein. Its inhibition in endothelial cells reduces proliferation and tube formation. However, inhibition of YAP1 leads to an increase in the release of exosomes containing MALAT1 into the tumor microenvironment. Exosome transfer of MALAT1 to hepatic cells increase invasion and migration via the activation of ERK1/2 signaling.	[91]
	Colorectal cancer(CRC)	lncRNA RPPH1	lncRNA RPPH1 is upregulated in CRC tissues associated with advanced TNM stages and poor prognosis. RPPH1 promote CRC metastasis in vitro and in vivo by inducing EMT of CRC cells via interacting with β-III tubulin (TUBB3) CRC cell-derived exosomes transport RPPH1 into macrophages mediating M2 polarization, which promotes metastasis and proliferation in CRC cells.	[92]
	Lung cancer	miR-499a-5p	MiR-499a-5p was upregulated in highly metastatic lung cancer cell line and in their exosomes promoting cell proliferation, migration and EMT via mTOR pathway.MiR-499a-5p knockdown suppress these processes in vitro supporting the potential diagnostic and therapeutic value of cancer-derived exosome miR-499a-5p.	[93]
	Glioma	miR-1246	Hypoxic glioma-derived exosomes (H-GDE) induce M2 macrophage polarization, which promotes glioma proliferation, migration and invasion in vitro and in vivo.MiR-1246 is the most abundant in H-GDE, and in the CSF of GBM patients, but its expression decreases after tumor resection. M2 macrophage polarization in H-GDE is mediated by miR-1246 targeting of TERF2IP to activate STAT3 and inhibit the NF-κB signaling pathways.	[94]
	Clear cell renal cell carcinoma (ccRCC)	ApoC1	ApoC1 overexpression in ccRCC cells is related to invasion, poor survival and EMT induction, thus promoting metastasis. In contrast, ApoC1 downregulation inhibits these effects. Transfer of exosome ApoC1 from ccRCC to endothelial cells promotes metastasis by activation of STAT3. This metastatic potential is suppressed by inhibition of DPP-4.	[95]

Legend: FOXO3a, The Forkhead box O-3a; GC, Gastric cancer; c-MYB, MYB proto-oncogene transcription factor; VEGF, Vascular Endothelial Growth Factor; BAMBI, BMP and Activin Membrane Bound Inhibitor; PTEN, Phosphatase and tensin homolog; AKT, serine-threonine protein kinase; TSGA10, Testis-Specific 10; CAFs, Cancer-associated fibroblasts; FGF2, Fibroblast Growth Factor 2; MMP9, Matrix Metallopeptidase 9; OC, Ovarian cancer; RCAN1.4, Regulator of Calcineurin 1 Isoform 4; RAMP2-AS1, RAMP2 Antisense RNA 1; HUVECs, Human umbilical vein endothelial cells; CSRP2,Cysteine And Glycine Rich Protein 2; OSCC, Oral squamous cell carcinoma; AXL, AXL Receptor Tyrosine Kinase; GSK-3β, Glycogen Synthase Kinase 3 Beta; CDX2, Caudal Type Homeobox 2; HOXA5, Homeobox A5; EMT, Epithelial-Mesenchymal Transition; TGF-β, Transforming Growth Factor Beta; MALAT1, Metastasis Associated Lung Adenocarcinoma Transcript 1; ERK1/2, The extracellular signal-regulated kinase 1/2; YAP1, Yes 1 Associated Transcriptional Regulator; RPPH1, Ribonuclease P RNA Component H1; TNM, Tumor, Node, Metastases; GBM, Glioblastoma; TERF2IP, TERF2 Interacting Protein; ApoC1, Apolipoprotein C1; STAT3, Signal Transducer And Activator of Transcription 3.

**Table 2:** Pre-clinical and clinical trials using exosomes for cancer therapy.

Clinical Approach	Source of Exosomes	Clinical and Pre-Clinical Trial Features	Reference or Clinical Trials
Extracellular vesicles-based cancer vaccine	Dendritic cell	Combined Immunotherapy using metronomic cyclophosphamide followed by vaccination with tumor antigen-loaded dendritic cell-derived exosomes to inhibit Treg cell functions and restore T and NK cell effector functions while activating innate and adaptive immunity.	[107,108,109,110,111] NCT01159288
	T cell	A novel HER2 specific exosome/T-cell vaccine using polyclonal CD4+ T cells up taking exosomes released by HER2-specific dendritic cells may provide a new therapeutic alternative for patients with trastuzumab-resistant HER2+ breast cancer.	[112,113]
	Tumor cells	Prostate cancer cell-derived exosomes were used to prepare a vaccine anchoring an IFN-γ fusion protein on the surface of prostate cancer cell-derived exosomes, which retained its bioactivity and significantly inhibited tumor growth and prolonged the survival time of mice with prostate cancer.	[114]
Tumor-associated or cell lines exosomes	Tumor cells	Exosomes secreted by diffuse large B cell lymphoma (DLBCL) induce apoptosis and upregulation of PD-1 in T cells. However, dendritic cells after being pulsed with DLBCL exosomes can stimulate T cells, increase IL-6 and TNFα expression and decrease the production IL-4 and IL-10. In another study, exosomes derived from heat-shocked mouse B lymphoma cells contained more HSP60 and HSP90, MHC I, MHC II, CD40, CD86, RANTES and IL-1β, which induce phenotypic and functional maturation of dendritic cells and activate CD8+ T cells to produce an antitumor effect	[115,116]
	Natural killer cell-derived exosomes	NK cell markers such as NKG2D, CD94, perforin, granzymes and CD40L are expressed in NK-derived exosomes, along with other molecules involved in cytotoxicity, homing, cell adhesion and immune activation, suggesting that NK exosomes might be potentially exploited in support of cancer therapy.	[117,118]
	CAR-T cell/CTL-derived exosomes	CAR-T cells release exosomes that carry CAR and express a high level of cytotoxic molecules capable of inhibiting tumor growth. They do not express PD1 and have cytotoxic activity in vitro. The presence of CTL surface membrane molecules in CTL-derived exosomes ensures the unidirectional delivery of the lethal hit to targeted tumor cells.	[119,120]
	Mesenchymal stromal cells-derived exosomes	To determine the best dose and side effects of exosomes derived from mesenchymal stromal cells loaded with KrasG12D siRNA for the pancreatic cancer treatment.	NCT03608631
Genetically Engineered	Antibodies on exosomes	Synthetic multivalent antibodies retargeted exosomes (SMART-Exo) were genetically modified for displaying two distinct types of monoclonal antibodies on the exosome surface. They can simultaneously target tumor-associated human EGFR and T-cell surface CD3 receptor redirecting and activating T cells which exhibit a highly potent and specific antitumor activity against EGFR-expressing cancer cells.	[121,122]
Natural product	Therapeutic effect of plant exosomes	To determine the effect of exosome-delivered curcumin on immune modulation, cellular metabolism and phospholipid profile in normal colon tissue and colon tumors of newly diagnosed colon cancer patients who are undergoing surgery. To investigate the ability of plant (grape) exosomes to prevent oral mucositis associated with chemo-radiation treatment of head and neck cancer. Fruit-derived exosomes and curcumin should not generate any side-effects.	[123] NCT01294072, NCT01668849
Chemical drugs	Exosome-based drug delivery platform for chemotherapeutic agents	Exosomes released by autologous macrophages loaded with paclitaxel (PTX) upon ultrasound treatment showed efficacy in the treatment of multidrug resistant cancer cells. This system may transport other chemotherapeutic agents in the future.	[103,104,124]
		Purified exosomes from immature DCs (imDCs) loaded with doxorubicin (Dox) via electroporation, showed highly efficient targeting and Dox delivery to αv integrin-positive breast cancer cells	
		Therapeutic strategy of codelivering genes and chemotherapeutic drugs, A15-Exosomes coloaded with Doxorubicin and Cho-miR159 induced synergistic therapeutic effects in MDA-MB-231 cells by effectively silenced the TCF-7 gene improving anticancer effects, without adverse effects	

Legend: HER2, Erb-B2 Receptor Tyrosine Kinase 2; CD4, T-Cell Surface Glycoprotein CD4; HSP60, Heat Shock Protein 60; HSP90, Heat Shock Protein 90; MHC I, Class I Major Histocompatibility Complex; MHC II, Class II Major Histocompatibility Complex; CD40, CD40 Molecule; CD86, CD86 Molecule; RANTES, Regulated upon Activation, Normal T Cell Expressed and Presumably Secreted; IL-1β, Interleukin 1 Beta; NKG2D, Receptor natural killer group 2, member D; CD90, CD90 molecule; CD40L, CD40 molecule ligand; CTL, Cytotoxic T cells; KRAS, KRAS Proto-Oncogene, GTPase; siRNA, small interfering RNA.

## Data Availability

No new data were created or analyzed in this study. Data sharing is not applicable to this article.

## Abbreviations

MVBs	Multivesicular bodies
TEX	Tumor-derived exosomes
DC	Dendritic cell
ILVs	Intraluminal vesicles
ESCRT	Endosomal distribution complex required for transport
APC	Antigen presenter cell
NK	Natural killer
TL	T lymphocyte
Treg	T-lymphocyte regulator
Breg	B-lymphocyte regulator
MHC	Major histocompatibility complex
TSPAN	Tetraspanins
LPBA:	Lysobisphosphatidic acid
ICAM:	Intercellular adhesion molecules
TCR	T-cell receptor
FasL	Fas Ligand
NKG2D	NKG2D: Natural killer group 2D receptor
CTLA-4	CTLA-4: Cytotoxic T-lymphocyte-associated antigen
PD1	Programmed Death 1
PD-L1	Programmed Death-ligand 1
GAPDH	Glyceraldehyde-3-phosphate dehydrogenase
TME	Tumor microenvironment
LncRNA	Long noncoding RNA
miRNA or miR	MicroRNA
circRNA	Circular RNA
BMDCs	Bone marrow-derived DCs
TLR	Toll-like receptors
MDSC	MDSC: Myeloid-derived suppressor cell
HLA	Human leukocyte antigen
TGF	Transforming growth factor
FGF	Fibroblast growth factor
PGE2	Prostaglandin 2
INF	Interferon
TNF	Tumor necrosis factor
TNFR	Receptor for tumor necrosis factor
MALAT-1	Long non-coding RNA metastasis-associated lung adenocarcinoma transcript 1
POU3F3	Long noncoding RNA POU class 3 homeobox 3
TAM	Tumor-associated macrophage
CAF	Cancer-associated fibroblast
EMT	Epithelial-mesenchymal transition
